# The interaction between mental resilience and insomnia disorder on negative emotions in nurses in Guangdong Province, China

**DOI:** 10.3389/fpsyt.2024.1396417

**Published:** 2024-07-29

**Authors:** Ningjing Zhan, Yixuan Xu, Jiangfeng Pu, Waner Wang, Zhanghao Xie, Huigen Huang

**Affiliations:** ^1^ Guangdong Mental Health Center, Guangdong Provincial People’s Hospital (Guangdong Academy of Medical Sciences), Southern Medical University, Guangzhou, China; ^2^ Department of Nursing, Guangdong Pharmaceutical University, Guangzhou, China; ^3^ Department of Nursing, Shantou University Medical College, Shantou, China; ^4^ Department of Nursing, Southern Medical University, Guangzhou, China

**Keywords:** nurses, psychological resilience, insomnia, negative emotions, correlation

## Abstract

**Objective:**

To investigate the current status of psychological resilience, insomnia and negative emotions among nurses in Guangdong Province, China. And to explore the effects of the interaction between psychological resilience and insomnia on negative emotions.

**Methods:**

A cross-sectional survey method was used to select 1874 nurses in Guangdong Province from February 2023 to April 2023 for the survey. The Chinese version of the Psychological Resilience Scale (PRS), Insomnia Severity Index (ISI), Depression Anxiety and Stress Scale-21(DASS-21)were used in this study. We use SPSS 26.0 for statistical analysis. A simple effect analysis was established to analyze the association between psychological resilience, insomnia and negative emotions by using psychological resilience, insomnia and the interaction term.

**Results:**

The overall PRS and ISI scores were (59.79 ± 17.29) and (9.83 ± 5.97). The scores of DASS-21 each dimension were (8.19 ± 9.02) for depression emotion,(7.93 ± 7.66) for anxiety emotion and (10.58 ± 9.77) for stress emotion. The results of the interaction effect analysis indicated that psychological resilience and insomnia exhibited statistically significant differences in the between-subjects effect test for depression emotion (r2 = 0.136, P<0.01), anxiety emotion (r2 = 0.127, P<0.01), and stress emotion (r2 = 0.142, P<0.01).

**Conclusion:**

The nurses exhibited moderate levels of psychological resilience. Most of them have varying degrees of insomnia, with majority being classified as mild insomnia. Additionally, they demonstrated varying degrees of negative emotions. There was a negative correlation between psychological resilience and insomnia. Psychological resilience was negatively correlated with depression, anxiety and stress emotions. Insomnia was positively correlated with depression, anxiety and stress emotions. Furthermore, the interaction between psychological resilience and insomnia had an effect on all three dimensions of negative emotions. Consequently, hospital administrators may implement efficacious intervention strategies such as cognitive behavioral therapy and improving workplace climate in a timely manner according to the different psychological tolerance and severity of insomnia of nurses in order to reduce the occurrence of negative emotions among nurses and improve their mental health.

## 1 Introduction

The ‘Healthy China 2030’ Planning Outline, issued by the State Council of China, emphasizes the need to strengthen the construction and standardized management of the mental health service system. It also aims to improve the prevention and treatment of common mental disorders, such as depression and anxiety, and the level of intervention in identifying psychological and behavioral problems. Additionally, it calls for increased efforts to detect psychological problems in key populations and provide timely intervention (1). As the largest professional group among medical practitioners, nurses have a unique occupational characteristics. Their work is often long-term and overloaded, with highly tense working conditions, irregular schedules, complex interpersonal conflicts, and frequent inspections and examinations (2). These factors have contributed to greater psychological pressure on nurses, making them a high-risk group for mental health problems. Furthermore, aggressive and harmful actions pose physical and safety hazards to healthcare professionals, while also directly impacting their mental health.

There is 71.2% of medical personnel have anxiety symptom scores higher than the clinical threshold, while 31.3% have anxiety, 26.8% are in a state of depression, and 36.7% have post-traumatic stress disorder[PTSD] (3). In China, more than 80% of medical personnel experience anxiety or depression, and 49.9% have symptoms of insomnia (4). Nurses’ emotional labor factors, such as anxiety, depression, and burnout, are associated with burnout and positively correlated with turnover intention (5). The departure of nurses from hospitals not only increases hospitals’ recruitment and training costs (6), but also hinders the improvement of nursing care quality and the stable construction of the nursing team (7). Administrators are increasingly recognizing the importance of addressing mental health issues among nurses. In order to improve mental health literacy and facilitate early detection and intervention of psychological problems in key groups, it is essential to establish mental health service mechanisms and carry out related activities.

Previous studies have primarily focused on physical and mental health promotion services for patients, neglecting the needs of nurses who are also at a higher risk of developing psychological problems such as anxiety, depression, and insomnia (8–10). It revealed that 40.58% of nurses experienced insomnia disorders, 36.71% had symptoms of anxiety, and 31.88% were in a state of depression in Poland (11). During the epidemic, Holton (12) found that nurses in Australia had higher levels of anxiety than doctors. It was also established that 41.8% of nurses had symptoms of anxiety and 55.5% were in a state of depression in 243 hospitals in 30 provinces in China (13).

During the Coronavirus 2019 (COVID-19) pandemic, nurses were asked to take extra shifts, work late, and skip breaks all in a continuously high-stress environment and worked under a tremendous amount of pressure (14). The pandemic affected the psychological health of nurses. Numerous nurses faced mental health complications associated with quarantine such as psychological distress and fear. The continuous stress nurses are faced, triggered post-traumatic stress symptoms, poor service delivery, suicide ideation, and suicide attempts (15). The high work pressure and workload, uncertainty about a poorly known and deadly disease, dehumanized healthcare working conditions in protective personal equipment, shorter time for social interactions with patients, numerous deaths, and family visit bans contributed to the psychological suffering of nurse during COVID-19 pandemic (16).

Psychological resilience is the process by which an individual copes and adapts positively when experiencing adversity, trauma, or stress (17). It is the ability to bounce back in the face of difficult experiences. Psychological resilience is considered a necessary condition for nurses to effectively regulate physical and psychological impairments, adapt to workplace stress and challenges, and recover quickly (18). Psychological resilience is influenced by both innate individual differences and environmental factors such as education and training. Petzold M.B (19) pointed out that individuals with lower psychological resilience are at a higher risk of developing anxiety and depression.

The sleep problems may be associated with other disorders, such as PTSD, depression, anxiety in a bidirectional relationship (20). Insomnia is highly prevalent in clinical practice, occurring in up to 50% of primary care patients. Insomnia can present independently or alongside other medical conditions or mental health disorders and is a risk factor for the development and exacerbation of other disorders if not treated (21). Frequent night shifts can disrupt the biological clock of clinical nurses, leading to negative impacts on their work and personal lives, as well as their sleep quality. Poor sleep quality can also have negative effects on their physical and mental health, potentially leading to anxiety and depression. It is important to note the close relationship between sleep and negative emotions (22). Previous studies have demonstrated a positive correlation between anxiety and depression with the sleep quality of clinical nurses (23).

Drawing from the Job Demands-Resources Model[JD-R], the characteristics of any job can be divided into job requirements and job resources. Job requirements are “negative factors” that consume individual energy in work, such as work overload, time pressure, job insecurity and so on. Job resources are “positive factors” in work, which can promote the realization of work goals, reduce work requirements and related psychological and physiological costs, or promote personal growth, learning and development (24). Due to the strong professionalism of clinical nursing work and frequent night shifts lead to the disturbance of the biological clock, it not only causes great trouble to the work and life of nurses, but also seriously affects the sleep quality of nurses (25). Low sleep quality would affect the physical and mental health of nurses, resulting in anxiety and depression (22). Psychological resilience is an important protective factor for individual mental health, and plays a key role when individuals face adversity and pressure (26). Nurses with high psychological resilience may encourage themselves, adjust themselves to cope with work pressure, reduce work tension, and are more inclined to deal with environmental stress events in a positive way, thus having a higher sense of work accomplishment.

The study investigated the mechanisms underlying insomnia disorder and the psychological resilience of nurses in the context of negative emotions. The findings may provide a theoretical basis for managers to understand the psychological needs of nurses and, to manage their human resources, to promote excellence in healthcare, as well as facilitate the implementation of measures for quality healthcare services.

## 2 Materials and methods

### 2.1 Design, setting and participants

We conducted a cross-sectional survey from February 2023 to April 2023 using snowball and convenience sampling methods to select a total of 1,874 nurses from hospitals of all levels in Guangdong Province, China as study subjects. Inclusion criteria: (1) registered nurses with licensure; (2) age ≥18 years old, engaged in clinical nursing for more than half a year; (3) informed consent and voluntary participation in this study. Exclusion criteria: (1) those who were on vacation for more than 3 months or more (e.g., studying abroad); (2) those who suffered a major life or work event in 3 months (e.g. experienced natural disasters such as earthquakes and floods).

### 2.2 Data collection

We used a unified guideline to inform the purpose, content and significance of the survey and the researcher’s contact information, and distributed the electronic questionnaire or QR code anonymously to members of the Guangdong Nursing Association through Wenjuanxing (a professional questionnaire collection website in China, https://www.wjx.cn), which was forwarded to nurses in each clinical department. Nurses can click on the cell phone to fill out the electronic questionnaire directly for anonymous answering. All options of the questionnaire had to be completed before submitting the uploaded results. If there were any questions about the completion process, respondents could ask the researcher at any time.

### 2.3 Ethical considerations

This study was approved by the Ethics Committee of Guangdong Provincial People’s Hospital (approval number: KY-Z-2020-25-652-03). Before data collection, the purpose of the study was briefly explained to the respondents and their consent was obtained before proceeding. Once they are willing to take part in the research, informed consent was acquired. The respondents had the right to withdraw from the study at any time. All the information in the study was restricted to be used only for the present study and it was promised that all the personal information of the respondents would never be divulged.

### 2.4 Measurements

#### 2.4.1 Variables related to demographic characteristics

A questionnaire was created to gather demographic and sleep-related information. The questionnaire consisted of fourteen items, including gender, age, marital status, education level, years of work experience, professional title, mode of appointment, hospital level, region of workplace, department, weekly hours of overtime, average monthly salary, and number of night shifts per month.

#### 2.4.2 Psychological resilience

To assess the psychological resilience of nurses, we utilized the Chinese version of the Psychological Resilience Scale (PRS) translated and revised by Chinese scholars Xiaonan Yu and Jianxin Zhang (27). The PRS comprises 25 questions and measures three dimensions of resilience: strength, optimism, and resilience. The concept of resilience was measured through 13 entries, such as the ability to adapt to change. Strength was measured through 8 entries, such as the ability to maintain focus and think carefully when experiencing stress. Optimism was measured through 4 entries, such as feeling in control of one’s life. The measurement scale used was a 5-point Likert scale, with 0 indicating not at all, 4 indicating almost always, and a total score of 0-100. Higher scores indicate better psychological resilience.

#### 2.4.3 Severity of insomnia

To measure the severity of insomnia in nurses, the Chinese version of the Insomnia Severity Index (ISI) translated and adapted by Chinese scholar Li Enze (28) was utilized. The scale comprises of 7 items, such as ‘How distressed do you feel about your current sleep problems?’, and is rated on a 5-point Likert scale ranging from 0 (not at all impaired) to 4 (extremely impaired). The scoring system ranged from 0 to 28, with 0-7 indicating no insomnia, 8-14 indicating mild insomnia, 15-21 indicating moderate insomnia, and 22-28 indicating severe insomnia. The scale demonstrated good reliability among the group of nurses, with a Cronbach’s alpha of 0.843.

#### 2.4.4 Negative emotions

The study utilized the Depression-Anxiety-Stress Self-Rating Scale Lite(DASS-21) to evaluate negative emotions. The scale comprises 21 entries divided into three dimensions: seven entries for each of the depression emotion dimension (e.g., feeling completely unable to be positive and optimistic), the anxiety emotion dimension (e.g., experiencing dryness in the mouth), and the stress emotion dimension (e.g., difficulty in calming down and resting). The scale ranges from 0 to 3. A score of 0 indicates that the option does not fit the reality at all, while a score of 1 indicates that the option fits the reality to some extent or some of the time. A score of 2 indicates that the option fits the reality to a great extent or most of the time, and a score of 3 indicates that the option fits the reality very well. To obtain the score for each scale, sum the items and multiply by a factor of 2. A score of 10 or higher on the depression subscale, 8 or higher on the anxiety subscale, and 15 or higher on the stress subscale indicates the presence of corresponding negative emotions (29).

### 2.5 Data analysis

The data were analyzed using IBM SPSS 26.0. Continuous variables that were normally distributed were expressed as means and standard deviations, while count data were expressed as percentages. The nurses’ scores for psychological resilience, insomnia severity index, depression emotion, anxiety emotion, and stress emotion were described using means and standard deviations. Percentages were used to describe the incidence of insomnia problems and negative emotions, including depression, anxiety, and stress emotions, among nurses. Chi-square tests were used to compare groups of different genders and ages. Univariate analyses were conducted to determine the variables to be included in the multivariate analyses. A logistic regression model was used to analyze the effect of negative emotions based on the stratification of psychological resilience and insomnia severity, after correcting for other confounders.

## 3 Results

### 3.1 Participants characteristics

A total of 1923 questionnaires were distributed in this study. Out of this figure, 36 questionnaires were excluded and the remaining 1874 valid questionnaires were recovered with a valid recovery rate of 98 percent. The questionnaires were obtained from 97 hospitals at different levels and areas in Guangdong Province, China. The average age of nurses in this study was (33.73 ± 8.16) years old, with a range of 19-54. The proportion of male and female nurses was 15% and 85% respectively. In the analyzed data, 70% were married, 60.2% had a bachelor’s degree, 24.8% had 6-10 years of working experience, and 39.5% were nurse practitioners. **Table 1** shows additional relevant descriptive data.

**Table 1:** Demographic characteristics of participants (N=1874).

Variable	Category	N	%
Gender	Male	281	15.0
	Female	1593	85.0
Age(years)	≤25	292	15.6
	26-35	916	48.9
	36-45	448	23.9
	>45	218	11.6
Marital status	married	1312	70.0
	unmarried	522	27.9
	divorced or separated	36	1.9
	bereaved of one’s spouse	4	0.2
Highest level of nursing education	vocational secondary school degree	63	3.4
	Associate degree	660	35.2
	bachelor’s degree	1128	60.2
	Master’s degree or above	23	1.2
Years of nursing experience(years)	<1	79	4.2
	1-5	332	17.7
	6-10	464	24.8
	10-15	433	23.1
	>15	566	30.2
Professional title	nurse	437	23.3
	senior nurse	741	39.5
	nurse practitioner-in-charge	558	29.8
	Associate Chief Nurse	124	6.6
	Associate Nurse	14	0.7
form of employment	Contract nurses	1134	60.5
	Staff nurses	675	36.0
	Personnel agency nurses	53	2.8
	Labor Dispatch Nurses	12	0.6
Hospital level	tertiary hospital	1341	71.6
	Level II hospitals	401	21.4
	Level I hospitals	124	6.6
	private hospital	8	0.4
Hospital Locations	The region in the east of Guangdong Province	126	6.7
	The region in the west of Guangdong Province	626	33.4
	The region in the north of Guangdong Province	331	17.7
	Pearl River Delta Region of Guangdong Province	791	42.2
unit (e.g. intensive care unit)	Internal medicine	444	23.7
	surgery	328	17.5
	obstetrics	188	10.0
	pediatric	64	3.4
	ICU	109	5.8
	operation theatre	148	7.9
	outpatient and emergency	286	15.3
	the rest	307	16.4
Overtime hours per week(hours)	T ≤ 5	967	51.6
	5<T ≤ 10	574	30.6
	10<T ≤ 15	198	10.6
	T>15	135	7.2
Monthly mean wag(yuan)	<5000	428	22.8
	5000-10000	1055	56.3
	10001-15000	282	15
	15001-20000	81	4.3
	>20000	28	1.5
Frequency of night shifts per month	0-2	653	34.8
	3-4	549	29.3
	5-6	448	23.9
	7-8	137	7.3
	≥9	87	4.6

### 3.2 Descriptions and construct validity of variables

The study analyzed the psychological resilience, insomnia severity, and negative emotions scores of 1874 nurses. The psychological resilience score was (59.79 ± 17.29), while the insomnia severity index score was (9.83 ± 5.98). The scores for depression emotion, anxiety emotion, and stress emotion were (8.19 ± 9.02), (7.93 ± 7.66), and (10.58 ± 9.77), respectively. In order to ascertain that the scales used in this study are reliable, we conducted a series of statistical tests to assess the internal consistency and construct validity of the three scales. These tests included the calculation of Cronbach’s coefficients, KMO values and Barlett’s test results. **Table 2** shows the current status of PRS, ISI and DASS-21 among nurses in Guangdong Province, China, and their reliability values.

**Table 2:** Means(M), Standard Deviations (SD) and reliability tests among study variables (N = 1874).

Scale	Dimension	*M* ± *SD*	Cronbach‘s alpha	KMO values	Bartlett test		
					approximate chi-square value	*df*	*p*
Psychological Resilience Scale	Resilience	32.12 ± 9.17	0.96	0.97	33196.01	300	<0.01
	Strength	18.19 ± 6.05					
	Optimism	9.49 ± 3.30					
Insomnia Severity Index	/	9.83 ± 5.98	0.90	0.88	8010.06	21	<0.01
Depression-Anxiety-Stress Self-Rating Scale	Depressed emotion	8.19 ± 9.02	0.97	0.98	33853.72	210	<0.01
	Anxious emotion	7.93 ± 7.66					
	Stressful emotion	10.58 ± 9.77					

### 3.3 The relationship between psychological resilience, insomnia and negative emotions in nurses

Pearson correlation analysis revealed a negative correlation between the dimensions of negative emotions and psychological resilience (*P*< 0.01) and a positive correlation between the dimensions of negative emotions and insomnia disorder (*P*< 0.01). The negative mood dimensions exhibited a weak correlation with psychological resilience, with anxiety demonstrating the lowest correlation at -0.094. The correlations between the three dimensions and insomnia disorder, from highest to lowest, were 0.601 for the stress dimension, 0.584 for the anxiety dimension, and 0.541 for the depression dimension, which were all greater than 0.5, indicating strong correlations. Other relevant coefficients are specified in **Table 3**.

**Table 3:** Correlation analysis of mental resilience, insomnia disorder and negative emotion.

	Variable	1	2	3	4	5	6	7	8
1	psychological resilience	-							
2	resilience dimension	0.958**	-						
3	Strength dimension	0.940**	0.825**	-					
4	Optimism dimension	0.871**	0.748**	0.820**	-				
5	insomnia severity index	-0.067**	-0.059*	-0.056*	-0.087**	-			
6	Depressed emotion dimension	-0.131**	-0.118**	-0.119**	-0.143**	0.541**	-		
7	anxious emotion dimension	-0.094**	-0.085**	-0.084**	-0.102**	0.584**	0.842**	-	
8	stressful emotion dimension	-0.120**	-0.111**	-0.111**	-0.120**	0.601**	0.885**	0.875**	-

*P < 0.05, ** P < 0.01.

### 3.4 Factors influencing insomnia disorder in nurses

The results of univariate analysis showed that the differences in insomnia severity index scores for age, marital status, job title, location of working hospital, department, weekly overtime hours, average monthly salary, and number of night shifts per month were statistically significant (*P*<0.05). The results are shown in **Table 4**.

**Table 4:** Univariate analysis of nurses’ insomnia severity index.

Variable	Category	N	ISI Scores	t/*F*	*P*
Gender	Male	281	9.50 ± 6.01	-0.995	0.32
	Female	1593	9.89 ± 5.97		
Age(years)	≤25	292	10.08 ± 5.817	2.666	0.046*
	26-35	916	10.13 ± 6.06		
	36-45	448	9.37 ± 5.70		
	>45	218	9.18 ± 6.33		
Marital status	married	1312	9.65 ± 5.92	3.125	0.025*
	unmarried	522	10.40 ± 6.05		
	divorced or separated	36	8.03 ± 6.55		
	bereaved of one’s spouse	4	8.75 ± 4.92		
Highest level of nursing education	vocational secondary school degree	63	9.86 ± 6.52	0.806	0.49
	Associate degree	660	10.04 ± 5.92		
	bachelor’s degree	1128	9.73 ± 5.96		
	Master’s degree or above	23	8.43 ± 6.91		
Years of nursing experience(years)	<1	79	9.73 ± 6.02	0.612	0.654
	1-5	332	10.01 ± 5.64		
	6-10	464	10.05 ± 6.30		
	10-15	433	9.87 ± 5.95		
	>15	566	9.52 ± 5.92		
Professional title	nurse	437	9.94 ± 5.69	3.294	0.011*
	senior nurse	741	10.04 ± 6.29		
	nurse practitioner-in-charge	558	9.84 ± 5.79		
	Associate Chief Nurse	124	8.64 ± 5.64		
	Associate Nurse	14	5.57 ± 5.84		
form of employment	Contract nurses	1134	9.87 ± 6.01	1.124	0.338
	Staff nurses	675	9.68 ± 5.90		
	Personnel agency nurses	53	10.15 ± 6.53		
	Labor Dispatch Nurses	12	12.67 ± 4.19		
Hospital level	tertiary hospital	1341	9.84 ± 5.97	0.203	0.894
	Level II hospitals	1341	9.84 ± 5.97	0.203	0.894
	Level I hospitals	401	9.91 ± 5.90		
	private hospital	132	9.45 ± 6.34		
Hospital Locations	The region in the east of Guangdong Province	126	8.67 ± 5.95	3.073	0.027*
	The region in the west of Guangdong Province	626	9.54 ± 6.25		
	The region in the north of Guangdong Province	331	9.97 ± 5.71		
	Pearl River Delta Region of Guangdong Province	791	10.18 ± 5.84	3.213	0.002**
unit (e.g. intensive care unit)	Internal medicine	444	9.26 ± 5.90	3.213	0.002**
	surgery	328	10.34 ± 6.02		
	obstetrics	188	10.49 ± 6.46		
	pediatric	64	7.73 ± 5.40		
	ICU	109	10.85 ± 5.98		
	operation theatre	148	10.25 ± 6.04		
	outpatient and emergency	286	10.00 ± 5.82		
	the rest	307	9.41 ± 5.80		
Overtime hours per week(hours)	T ≤ 5	967	9.44 ± 5.73	3.807	0.010**
	5<T ≤ 10	574	10.05 ± 6.08		
	10<T ≤ 15	198	10.89 ± 6.36		
	T>15	135	10.10 ± 6.46		
Monthly mean wag(yuan)	<5000	428	10.38 ± 6.21	3.654	0.006**
	5000-10000	1055	9.60 ± 5.77		
	10001-15000	282	10.39 ± 6.38		
	15001-20000	81	8.77 ± 5.88		
	>20000	28	7.50 ± 4.86		
Frequency of night shifts per month	0-2	653	9.07 ± 5.85	4.725	0.001**
	3-4	549	10.03 ± 5.59		
	5-6	448	10.20 ± 6.19		
	7-8	137	10.72 ± 6.27		
	≥9	87	10.92 ± 7.06		

*P<0.05, **P<0.01.

### 3.5 Comparisons among different negative emotions

Among nurses, 42.16% (700/1874) had mild insomnia, 16.76% (314/1874) had moderate insomnia, and 3.73% (70/1874) had severe insomnia. **Table 5** shows statistically significant differences in the detection rates of depressive emotion, anxiety emotion, and stress emotion among nurses with different age, marital status, nursing age, title, appointment method, department, weekly overtime hours, average monthly salary, and number of night shifts per month (*P*< 0.05). Specifically, nurses belonging to the departments of surgery, ICU, and operation theatre, with weekly overtime hours greater than 15 hours, and with 9 or more night shifts per month, showed statistically significant differences (*P*< 0.05). A higher rate of stress, anxiety, and depressive symptoms were detected among nurses in the Pearl River Delta region compared to those in the eastern, western, and northern regions of Guangdong province (*P*< 0.05).

**Table 5:** Comparison of negative emotion detection rates by demographics and related variables (N=1874).

Variable	Category	n	depressed emotion		anxious emotion		stressed emotion	
			Number of detections. (YES)	χ2 values	Number of detections. (YES)	χ2 values	Number of detections. (YES)	χ2 values
Gender	Male	282	111(36.8%)	0.647	126(44.7)	0.242	83(29.4)	1.571
	Female	1593	587(39.4%)		737(46.3)		412(25.9)	
Age(years)	≤25	292	107(36.6)	22.733**	154(52.7)	27.321**	87(29.8)	15.915**
	26-35	919	382(41.7)		448(48.9)		267(29.1)	
	36-45	448	154(34.4)		191(42.6)		101(22.5)	
	>45	218	55(25.2)		70(32.1)		40(18.3)	
Marital status	married	1312	464(35.4)	8.849*	574(43.8)	11.745*	324(24.7)	7.877*
	unmarried	523	218(41.7)		273(52.2)		160(30.6)	
	Divorced or separated	36	13(36.1)		15(41.7)		9(25.0)	
	bereaved of one’s spouse (literary)	4	3(75)		1(25.0)		2(50.0)	
Highest level of nursing education	vocational secondary school degree	63	20(31.7)	3.339	29(46)	6.028	14(22.2)	5.254
	Associate degree	660	245(37.1)		313(47.4)		185(86.0)	
	bachelor’s degree	1129	428(37.9)		516(45.7)		294(26.0)	
	Master’s degree or above	23	5(21.7)		5(21.7)		2(8.7)	
Years of nursing experience(years)	<1	79	19(24.1)	28.883**	36(45.6)	23.452**	15(19.0)	14.049*
	1-5	332	145(43.7)		180(54.2)		104(31.3)	
	6-10	465	198(42.6)		230(49.5)		137(29.5)	
	10-15	433	164(37.9)		198(45.7)		114(26.3)	
	>15	566	172(30.4)		219(38.7)		125(22.1)	
Professional title	nurse	437	167(38.2)	19.536**	221(50.6)	24.018**	120(27.5)	19.323**
	senior nurse	742	306(41.2)		361(48.7)		226(30.5)	
	nurse practitioner-in-charge	558	192(34.4)		240(43)		125(22.4)	
	Associate Chief Nurse	124	32(25.8)		39(31.5)		24(19.4)	
	Associate Nurse	14	1(7.1)		2(14.3)		0(0.0)	
form of employment	Contract nurses	1135	466(41.1)	31.504**	564(49.7)	17.288**	326(28.7)	17.135**
	Staff nurses	675	200(29.6)		268(39.7)		144(21.3)	
	Personnel agency nurses	53	29(54.7)		26(49.1)		21(39.6)	
	Labor Dispatch Nurses	12	3(25.0)		5(41.7)		4(33.3)	
Hospital level	tertiary hospital	1342	496(37.0)	2.155	620(46.2)	0.210	342(25.6)	4.140
	Level II hospitals	401	157(39.2)		184(45.9)		120(29.9)	
	Level I hospitals	124	41(33.1)		55(44.4)		29(23.4)	
	private hospital	8	4(50.0)		4(50.0)		3(37.5)	
Hospital Locations	The region in the east of Guangdong	127	39(30.7)	9.575*	57(44.9)	1.446	31(24.4)	2.124
	The region in the west of Guangdong	626	219(35.0)		277(44.2)		156(24.9)	
	The region in the north of Guangdong	331	115(34.7)		157(47.4)		86(26.0)	
	Pearl River Delta Region of Guangdong Province	791	325(41.1)		372(47.0)		222(28.1)	
unit (e.g. intensive care unit)	Internal medicine	444	172(38.7)	20.977*	205(46.2)	18.633*	122(27.5)	15.317*
	surgery	329	137(41.6)		166(50.5)		104(31.6)	
	obstetrics	188	67(35.6)		99(52.7)		45(23.9)	
	pediatric	64	19(29.7)		26(40.6)		19(29.7)	
	ICU	109	52(47.7)		54(49.5)		30(27.5)	
	operating rooms	148	62(41.9)		75(50.7)		47(31.8)	
	outpatient and emergency	286	100(35.0)		123(43.0)		64(22.4)	
	the rest	307	89(29.0)		115(37.5)		64(20.8)	
Overtime hours per week(hours)	T ≤ 5	967	321(33.2)	19.962**	400(41.4)	20.550**	212(21.9)	29.302**
	5<T ≤ 10	574	226(39.4)		280(48.8)		160(27.9)	
	10<T ≤ 15	198	82(41.4)		109(55.1)		73(36.9)	
	T>15	136	69(50.7)		74(54.4)		50(36.8)	
Monthly mean wage(yuan)	<5000	429	174(40.6)	10.896*	221(51.5)	19.161**	124(28.9)	10.250*
	5000-10000	1055	371(35.2)		475(45)		273(25.9)	
	10001-15000	282	120(42.6)		135(47.9)		81(28.7)	
	15001-20000	81	27(33.3)		26(32.1)		15(18.5)	
	>20000	28	6(21.4)		6(21.4)		2(7.1)	
Frequency of night shifts per month	0-2	653	195(29.9)	33.479**	235(36.0)	43.003**	127(19.4)	34.387**
	3-4	549	215(39.2)		278(50.6)		155(28.2)	
	5-6	449	179(39.9)		226(50.3)		132(29.4)	
	7-8	137	60(43.8)		73(53.3)		43(31.4)	
	≥9	87	49(56.3)		51(58.6)		38(43.7)	
Psychological resilience level	Low psychological resilience	333	169(24.2)	72.901**	186(21.6)	47.648**	126(25.5)	47.685**
	Medium psychological resilience	839	348(49.9)		423(49.1)		234(47.4)	
	High psychological resilience	702	180(25.8)		253(29.4)		134(27.1)	
Insomnia severity	No insomnia	700	154(22.1)	186.169**	208(24.1)	166.264**	106(21.5)	205.857**
	Mild insomnia	789	304(43.6)		395(45.8)		191(38.7)	
	Moderate insomnia	314	181(26.0)		196(22.7)		142(28.7)	
	Severe insomnia	71	58(8.3)		63(7.3)		55(11.1)	

Detection rates in parentheses(), *P < 0.05, **P < 0.01.

#### 3.5.1 Interaction of psychological resilience and insomnia disorder on negative emotions

A multifactorial logistic regression model was developed to examine the relationship between the three symptoms of negative emotions (depression, anxiety, and stress) and two independent variables: psychological elasticity (low=2, medium=1, high=0) and insomnia severity (no=0, mild=1, moderate=2, severe=3). The results of the data analysis indicated that there was a significant interaction between psychological resilience and insomnia with the presence of depressed emotion (R^2^ = 0.136, *F* =13.073, *P*<0.01). Furthermore, there was a significant interaction between psychological resilience and insomnia with the presence of anxiety emotion (R^2^ = 0. 127).The results indicated that psychological resilience and insomnia interacted significantly with the presence of a stressful emotion (*F*=13.691, *P*<0.01). Furthermore, the interaction was found to be significant (adjusted R^2^ = 0.142, *F* = 11.083, *P* < 0.01). For further details on the results of the thematic effects tests, **Table 6** provides a comprehensive overview.

**Table 6:** A test of the between-subjects effect of negative emotions.

Independent variable	Implicit variable	*df*	*F*	*P*	R^2^
Psychological resilience*Insomnia	Depression emotion	6	13.073	*P*<0.010	0.136
Psychological resilience		2	4.923	0.007	
Insomnia		3	59.387	*P*<0.010	
Psychological resilience*Insomnia	Anxiety emotion	6	13.691	*P*<0.010	0.127
Psychological resilience		2	4.283	0.014	
Insomnia		3	56.9	*P*<0.010	
Psychological resilience*Insomnia	Stress emotion	6	11.083	*P*<0.010	0.142
Psychological resilience		2	11.487	*P*<0.010	
Insomnia		3	69.904	*P*<0.01	

*P < 0.05, **P < 0.01.

#### 3.5.2 Simple effects of psychological resilience and insomnia disorder on depression emotion

The results of the simple effects analysis indicated that nurses with severe, moderate, and mild insomnia disorders exhibited significantly higher levels of depression than nurses without insomnia (*p*<0.01). Furthermore, the depression emotion of those with severe and moderate insomnia was significantly higher than those with mild insomnia (*p*<0.01). In addition, the difference in depression emotion between moderate insomnia and severe insomnia was not statistically significant.

When the psychological resilience was at a medium level, the depression emotion was significantly higher in those with severe insomnia, moderate insomnia and mild insomnia than in those without insomnia (*p*<0.001). The difference was statistically significant. The depressive emotion was significantly higher in those with severe insomnia and moderate insomnia than in those with mild insomnia, and the difference being statistically significant.

The results indicated that depressed emotion was significantly higher in severe insomnia than in moderate insomnia. However, the differences in depressive emotion between the different levels of insomnia disorder were not statistically significant when psychological resilience was high. Further details can be found in **Table 7**.

**Table 7:** Simple effect analysis of depression emotion.

Psychological resilience	(I) Insomnia	(J) Insomnia	*M*	*SD*	95%*CI*	
					lower	upper
Low	NO	Mild	-0.285**	0.059	-0.441	-0.129
		Moderate	-0.509**	0.073	-0.702	-0.316
		Severe	-0.781**	0.124	-1.109	-0.453
	Mild	NO	0.285**	0.059	0.129	0.441
		Mild	-0.224*	0.071	-0.410	-0.037
		Severe	-0.496**	0.123	-0.820	-0.172
	Moderate	NO	0.509**	0.073	0.316	0.702
		Mild	0.224*	0.071	0.037	0.410
		Severe	-0.272	0.130	-0.616	0.071
	Severe	NO	0.781**	0.124	0.453	1.109
		Mild	0.496**	0.123	0.172	0.820
		Moderate	0.272	0.130	-0.071	0.616
Medium	NO	Mild	-0.300**	0.034	-0.388	-0.212
		Moderate	-0.501**	0.044	-0.618	-0.384
		Severe	-0.763**	0.077	-0.966	-0.560
	Mild	NO	0.300**	0.034	0.212	0.388
		Moderate	-0.201**	0.044	-0.316	-0.086
		Severe	-0.463**	0.077	-0.665	-0.261
	Moderate	NO	0.501**	0.044	0.384	0.618
		Mild	0.201**	0.044	0.086	0.316
		Severe	-0.262*	0.082	-0.478	-0.046
	Severe	NO	0.763**	0.077	0.560	0.966
		Mild	0.463**	0.077	0.261	0.665
		Moderate	0.262*	0.082	0.046	0.478
High	NO	Mild	0.069	0.039	-0.034	0.172
		Moderate	-0.086	0.052	-0.223	0.050
		Severe	-0.188	0.110	-0.478	0.102
	Mild	NO	-0.069	0.039	-0.172	0.034
		Moderate	-0.155*	0.051	-0.289	-0.020
		Severe	-0.257	0.110	-0.545	0.032
	Moderate	NO	0.086	0.052	-0.050	0.223
		Mild	0.155*	0.051	0.020	0.289
		Severe	-0.102	0.115	-0.404	0.200
	Severe	NO	0.188	0.110	-0.102	0.478
		Mild	0.257	0.110	-0.032	0.545
		Moderate	0.102	0.115	-0.200	0.404

*P < 0.05, **P < 0.01.

#### 3.5.3 Simple effects of psychological resilience and insomnia disorder on anxiety and stress emotion

The impact of psychological resilience and insomnia disorder on anxiety and stressful emotions was analyzed using **Table 8** and **Table 9**. The results were identical to those observed in depressed emotion, with statistically significant differences between different levels of insomnia disorder when psychological resilience was at low and medium levels. It is noteworthy that when the level of psychological elasticity was high, the prevalence of stressful emotion was significantly higher in individuals with severe insomnia than in those with mild insomnia (*p*<0.05). However, the differences in depressive emotion and anxiety emotion were not statistically significant.

**Table 8:** Simple effect analysis of anxiety emotion.

Psychological resilience	(I) Insomnia	(J) Insomnia	*M*	*SD*	95%*CI*	
					lower	upper
Low	NO	Mild	-0.374**	0.061	-0.536	-0.212
		Moderate	-0.582**	0.076	-0.782	-0.382
		Severe	-0.819**	0.129	-1.159	-0.479
	Mild	NO	0.374**	0.061	0.212	0.536
		Mild	-0.208*	0.073	-0.402	-0.014
		Severe	-0.445*	0.127	-0.781	-0.110
	Moderate	NO	0.582**	0.076	0.382	0.782
		Mild	0.208*	0.073	0.014	0.402
		Severe	-0.237	0.135	-0.593	0.118
	Severe	NO	0.819**	0.129	0.479	1.159
		Mild	0.445*	0.127	0.110	0.781
		Moderate	0.237	0.135	-0.118	0.593
Medium	NO	Mild	-0.323**	0.035	-0.414	-0.231
		Moderate	-0.468**	0.046	-0.589	-0.346
		Severe	-0.728**	0.080	-0.938	-0.518
	Mild	NO	0.323**	0.035	0.231	0.414
		Moderate	-0.145*	0.045	-0.264	-0.025
		Severe	-0.405**	0.079	-0.615	-0.196
	Moderate	NO	0.468**	0.046	0.346	0.589
		Mild	0.145*	0.045	0.025	0.264
		Severe	-0.260*	0.085	-0.484	-0.036
	Severe	NO	0.728**	0.080	0.518	0.938
		Mild	0.405**	0.079	0.196	0.615
		Moderate	0.260*	0.085	0.036	0.484
High	NO	Mild	0.031	0.040	-0.076	0.137
		Moderate	-0.012	0.054	-0.154	0.129
		Severe	-0.216	0.114	-0.516	0.084
	Mild	NO	-0.031	0.040	-0.137	0.076
		Moderate	-0.043	0.053	-0.182	0.096
		Severe	-0.247	0.114	-0.546	0.052
	Moderate	NO	0.012	0.054	-0.129	0.154
		Mild	0.043	0.053	-0.096	0.182
		Severe	-0.204	0.119	-0.517	0.109
	Severe	NO	0.216	0.114	-0.084	0.516
		Mild	0.247	0.114	-0.052	0.546
		Moderate	0.204	0.119	-0.109	0.517

*P < 0.05, **P < 0.01.

**Table 9:** Simple effect analysis of stress emotion.

Psychological resilience	(I) Insomnia	(J) Insomnia	M	SD	95%CI	
					lower	upper
Low	NO	Mild	-0.200*	0.054	-0.342	-0.058
		Moderate	-0.573**	0.067	-0.749	-0.398
		Severe	-0.829**	0.113	-1.126	-0.531
	Mild	NO	0.200*	0.054	0.058	0.342
		Moderate	-0.373**	0.064	-0.543	-0.204
		Severe	-0.629**	0.112	-0.923	-0.334
	Moderate	NO	0.573**	0.067	0.398	0.749
		Mild	0.373**	0.064	0.204	0.543
		Severe	-0.255	0.118	-0.567	0.057
	Severe	NO	0.829**	0.113	0.531	1.126
		Mild	0.629**	0.112	0.334	0.923
		Moderate	0.255	0.118	-0.057	0.567
Medium	NO	Mild	-0.148**	0.031	-0.228	-0.068
		Moderate	-0.397**	0.040	-0.504	-0.291
		Severe	-0.743**	0.070	-0.927	-0.558
	Mild	NO	0.148**	0.031	0.068	0.228
		Moderate	-0.249**	0.040	-0.354	-0.144
		Severe	-0.595**	0.070	-0.778	-0.411
	Moderate	NO	0.397**	0.040	0.291	0.504
		Mild	0.249**	0.040	0.144	0.354
		Severe	-0.346**	0.075	-0.542	-0.149
	Severe	NO	0.743**	0.070	0.558	0.927
		Mild	0.595**	0.070	0.411	0.778
		Moderate	0.346**	0.075	0.149	0.542
High	NO	Mild	0.035	0.036	-0.059	0.128
		Moderate	-0.037	0.047	-0.161	0.087
		Severe	-0.259	0.100	-0.522	0.004
	Mild	NO	-0.035	0.036	-0.128	0.059
		Moderate	-0.071	0.046	-0.194	0.051
		Severe	-0.294*	0.100	-0.556	-0.031
	Moderate	NO	0.037	0.047	-0.087	0.161
		Mild	0.071	0.046	-0.051	0.194
		Severe	-0.222	0.104	-0.497	0.052
	Severe	NO	0.259	0.100	-0.004	0.522
		Mild	0.294*	0.100	0.031	0.556
		Moderate	0.222	0.104	-0.052	0.497

*P < 0.05, **P < 0.01.

## 4 Discussion

In this study, the detection rates of depression, anxiety, and stress symptoms among nurses were 37.2%, 46%, and 21.1%, respectively, which were higher than the findings of Erdogan (30) among Turkish nurses (22.3%, 19.0%, and 14.3%), which may be related to the differences in social benefits and cultural backgrounds of nurses in different countries. Lower than the findings of Liu Min (31), who used the same scale on obstetric nurses in 20 hospitals in Wuxi City (42.4%, 61.7%, 35.7%), probably due to the fact that the subjects included in this study are nurses from different departments. Further, the clients of obstetric nursing are pregnant women and infants, and the overall level of stress is higher than that in other departments. It suggested that focus should be placed on special departments (e.g., obstetrics, pediatrics, intensive care, etc.) on the psychological situation of nurses.

In this study, nurses aged ≤25 years had the highest score for insomnia disorder. Perhaps, nurses in this age group are mostly at the stage of new recruitment, facing the transition from student status to nurse status, and adapting to a new rhythm of life and interpersonal relationships. Concurrently, nurses in this age group are more prone to developing insomnia disorders due to their incomplete grasp of the requisite professional knowledge in their field. The lowest level of insomnia disorder was observed in nurses over the age of 45. Nurses in this age group are highly skilled in their work, and the experience they have gained over the years allows them to face work emergencies with greater composure and less psychological distress, which in turn reduces the likelihood of insomnia disorder.

Almost all subcategories of medical workers perceive the stress related to the outbreak equally. Only long-career medical workers and ambulance service personnel reported needing psychological support less compared to other categories. This outcome may be explained by the high level of experience and long-standing training in stress management in long-career employees. On the other hand, emergency services personnel may be used to managing stressful situations, as they are part of a coordinated and ordered emergency response and have to constantly handle very high levels of stress (32). In terms of department, ICU nurses exhibited the highest level of insomnia, with a mean score of 10.85 ± 5.98. This result is lower than the mean score of 13.09 ± 5.54 reported by Khatony A (33) in their investigation of insomnia disorders among nurses in six hospitals in Iran. One possible explanation for this discrepancy is that the differences in the level of medical care and the environment in the two countries might have led to a greater number of stressors being faced by nurses in their work, possibly accounting for the reported higher levels of insomnia disorders. Furthermore, the noise pollution of ICUs, which utilize a multitude of medical devices, including cardiac monitors, ventilators, infusion pumps, suction machines and patients’ groans, could give rise to psychological stress and result in elevated levels of insomnia among nurses.

As the number of night shifts increases, the prevalence of insomnia among nurses also rises. Previous studies have demonstrated a correlation between the number of night shifts and the prevalence of insomnia disorder among nurses. Chung Y (34) found that nurses who work three or more consecutive night shifts are at an increased risk of developing insomnia disorder. Long-term night shifts disrupt the body’s circadian rhythm, which may result in alterations to the body’s hormone levels, leading to decreased immunity, endocrine disorders (35). These changes, in turn, may contribute to the onset of insomnia.

The results of the study showed that the negative emotions detection rate of nurses with insomnia symptoms was higher than that nurses without insomnia symptoms, and the highest negative emotion detection rate was found in nurses with mild insomnia status (43.6%, 43.8%, and 38.7%), and that the severity of nurses’ insomnia was an important predictor of negative emotion and shown a positive correlation between them. Insomnia is closely related to negative emotions such as depression and anxiety (36). Furihata R (37) found that poor sleep quality can have negative physical and mental health effects such as fatigue, burnout and depression, as well as increasing susceptibility to stress and the likelihood of mental health problems (38). Poor sleep quality has been found to be highly correlated with impaired cognitive function such as attention and memory, which can lead to poor performance and even compromise patient safety (39), while increasing susceptibility to stress and the likelihood of mental health problems (38). In addition, psychological resilience has been negatively associated with negative emotions in nurses, with the highest rate of negative emotion detection in nurses with moderate levels of psychological resilience. Psychological resilience plays an important role in the regulation of nurses’ physical and mental health as an individual protective resource that helps to prevent stress and trauma and to recover from these adverse events. Individuals with high levels of psychological resilience have been shown to be better able to recover from adverse events, to better manage their emotions and cognitions, and to maintain more stable and healthy physiological and psychological levels (40).

The interaction term between psychological resilience and insomnia disorder for nurses demonstrated significant between-subject effects on all three dimensions of negative emotions. This outcome suggests that the interaction term between different levels of psychological resilience and different degrees of insomnia disorder had different effects on negative emotions. As the level of nurses’ psychological resilience increased, the tendency of increasing depression when the level of insomnia disorder increased decreased. This pattern indicates that nurses’ psychological resilience can enhance nurses’ mental toughness in order to mitigate the adverse effects of insomnia disorder, and thus reduce negative emotions. Nursing managers may provide nurses with specialized psychological training to assist them in developing effective coping strategies for stress, frustration, and emotion management. Concurrently, a supportive work environment is established in order to facilitate nurses’ sense of being understood and cared for. Regular face-to-face communication with nurses, listening to their distress and feelings, and providing necessary support and assistance are effective methods for improving nurses’ psychological resilience and reducing their negative emotions.

At the same level of psychological resilience, different levels of insomnia disorders have the greatest effect on anxiety and the least effect on stress emotions. It can be posited that increased levels of insomnia disorders are more likely to exacerbate nurses’ anxiety. Consequently, it is imperative that managers perform their duties in a manner that effectively manages nurses’ anxiety in the workplace. Furthermore, it is of paramount importance that managers pay close attention to nurses’ mental health status, with the aim of detecting and identifying anxiety in a timely manner. The identification of signs of anxiety and the implementation of timely interventions such as cognitive behavioral therapy and improving workplace climate can be achieved through the observation of nurses’ behavioral performance, emotional changes and communication with nurses.

## 5 Strengths and limitations

Starting from psychological resilience and insomnia disorder, this study explores the impact of their interaction on negative emotions, which enricfies the theoretical research on mental resilience, insomnia disorder and negative emotions to a certain extent. In practice, these findings are important for improving the mental health of nurses and provide the basis for managers to intervene in nurses’ mental health.

There are some limitations in this study. Firstly, using purposive sampling in this study may restrict the generalizability of the research findings. In order to enhance the representativeness of the sample, 97 hospitals located across various regions of the Pearl River Delta, eastern, western and northern Guangdong Province were selected. Secondly, as this is a cross-sectional study, it only establishes associations between psychological resilience, insomnia disorders and negative emotions without inferring causality. In order to make up for this deficiency, future longitudinal studies need to be further confirmed.

### 5.1 Practical implications

This study analyzes the relationship between psychological resilience, insomnia disorders, and negative emotions, and explores the impact of the interaction between psychological resilience and insomnia disorders on negative emotions. Hospital managers can use this study to understand psychological resilience, insomnia disorders, and negative emotions, evaluate the mental health level of nurses, and provide timely psychological intervention based on different levels of psychological resilience and insomnia disorder status. The government can carry out corresponding measures to enhance the mental health level of nurses, reduce their willingness to resign, and stabilize the stability of the nursing team, which is of great practical significance. In future research, multi center and large sample surveys can be conducted to explore the differences in the psychological health status of nurses at different levels of economic development and regions, and to analyze the factors that affect the psychological health level of nurses from multiple perspectives.

## 6 Conclusion

Insomnia and psychological resilience are related to nurses’ negative emotional symptoms, and that insomnia and psychological resilience interact to influence nurses’ negative emotional symptoms. Based on the findings, there is the need to strengthen nurses’ sleep health education and psychological resilience development in real life, to actively conduct sleep hygiene counselling, and to timely intervene with nurses who already have sleep problems, which is an important and practical significance to improve their mental health and reduce symptoms of depression, anxiety, and stress.

## Data availability statement

The original contributions presented in the study are included in the article/supplementary material. Further inquiries can be directed to the corresponding author.

## Ethics statement

The Guangdong Provincial People’s Hospital Ethics Committee examined and authorized the studies involving human volunteers. To take part in this study, the patients/participants submitted their written, informed consent.

## Author contributions

NZ: Writing – original draft, Writing – review & editing. YX: Data curation, Writing – review & editing. JP: Data curation, Software, Writing – review & editing. WW: Writing – review & editing. ZX: Investigation, Writing – review & editing. HH: Project administration, Supervision, Writing – original draft, Writing – review & editing.

## References

[1] Outline of the "Healthy China 2030" Programme [EB/OL]. Available online at: www.gov.cn.

[2] Xin S Jiang W Xin Z. Changes in Chinese nurses' mental health during 1998-2016: A cross-temporal meta-analysis. Stress Health J Int Soc Invest Stress. (2019) 35:665–74. doi: 10.1002/smi.2907 31692200

[3] Hayat K Arshed M Fiaz I Afreen U Khan FU Khan TA. Impact of COVID-19 on the mental health of healthcare workers: A cross-sectional study from Pakistan. Front Public Health. (2021) 9:603602. doi: 10.3389/fpubh.2021.603602 33981657 PMC8107369

[4] Wang H Huang D Huang H Zhang J Guo L Liu Y. The psychological impact of COVID-19 pandemic on medical staff in Guangdong, China: a cross-sectional study. psychol Med. (2022) 52:884–92. doi: 10.1017/S0033291720002561 PMC7371926 32624037

[5] Kwon CY Lee B Kwon OJ Kim MS Sim KL Choi YH. Emotional labor, burnout, medical error, and turnover intention among South Korean nursing staff in a university hospital setting. Int J Environ Res Public Health. (2021) 18:10111. doi: 10.3390/ijerph181910111 34639412 PMC8507784

[6] Qian Y. A study of nurse retention strategies in tertiary care hospitals. Zhejiang China: Zhejiang University (2017), PhD dissertation.

[7] Schwartz R Sinskey JL Anand U Margolis RD. Addressing postpandemic clinician mental health : A narrative review and conceptual framework. Ann Internal Med. (2020) 173:981–8. doi: 10.7326/M20-4199 PMC7450528 32822206

[8] Li Z Li X Qin M Luo Z Yang W Lan Y. Analysis of psychological stress condition and influencing factors of nurses in infectious disease hospitals in epidemic prevention and control. Health Prof Educ. (2021) 23):106–8.

[9] Song Y Lai X Lin J. Mental health status and influencing factors of frontline medical staff in Wenzhou during the normalisation of epidemic prevention and control. J Wenzhou Med Univ. (2023) 53.02:166–73.

[10] Maharaj S Lees T Lal S. Prevalence and risk factors of depression, anxiety, and stress in a cohort of Australian nurses. Int J Environ Res Public Health. (2018) 16:61. doi: 10.3390/ijerph16010061 30591627 PMC6339147

[11] Rachubińska K Cybulska AM Sołek-Pastuszka J Panczyk M Stanisławska M Ustianowski P. Assessment of psychosocial functioning of polish nurses during COVID-19 pandemic. Int J Environ Res Public Health. (2022) 19:1435. doi: 10.3390/ijerph19031435 35162456 PMC8835236

[12] Holton S Wynter K Trueman M Bruce S Sweeney S Crowe S. Psychological well-being of Australian hospital clinical staff during the COVID-19 pandemic. Aust Health Rev Publ Aust Hosp Assoc. (2021) 45:297–305. doi: 10.1071/AH20203 33032681

[13] Li Y Fan R Lu Y Li H Liu X Kong G. Prevalence of psychological symptoms and associated risk factors among nurses in 30 provinces during the COVID-19 pandemic in China. Lancet regional Health Western Pacific. (2023) 30:100618. doi: 10.1016/j.lanwpc.2022.100618 36276987 PMC9576138

[14] Riedel B Horen SR Reynolds A Hamidian Jahromi A. Mental health disorders in nurses during the COVID-19 pandemic: implications and coping strategies. Front Public Health. (2021) 9:707358. doi: 10.3389/fpubh.2021.707358 34765579 PMC8575697

[15] Chidiebere Okechukwu E Tibaldi L La Torre G. The impact of COVID-19 pandemic on mental health of Nurses. Clin Ter. (2020) 171:e399–400. doi: 10.7417/CT.2020.2247 32901781

[16] Guessoum SB Moro MR Mallet J. The COVID-19 pandemic: do not turn the health crisis into a humanity crisis. Prim Care Companion CNS Disord. (2020) 22:20com02709. doi: 10.4088/PCC.20com02709 32678524

[17] Southwick SM Charney DS. The science of resilience: implications for the prevention and treatment of depression. Sci (New York N.Y.). (2012) 338:79–82. doi: 10.1126/science.1222942 23042887

[18] Dorris L. Considering resilience models in recovery from pediatric stroke. Eur J paediatric Neurol. (2020) 25:1. doi: 10.1016/j.ejpn.2020.02.008 32115368

[19] Petzold MB Bendau A Plag J Pyrkosch L Mascarell Maricic L Betzler F. Risk, resilience, psychological distress, and anxiety at the beginning of the COVID-19 pandemic in Germany. Brain Behav. (2020) 10:e01745. doi: 10.1002/brb3.1745 32633464 PMC7361063

[20] Salari N Khazaie H Hosseinian-Far A Ghasemi H Mohammadi M Shohaini S. The prevalence of sleep disturbances among physicians and nurses facing the COVID-19 patients: a systematic review and meta-analysis. Global Health. (2020) 16:92. doi: 10.1186/s12992-020-00620-0 32993696 PMC7522913

[21] Perlis ML Posner D Riemann D Bastien CH Teel J Thase M. Insomnia. Lancet. (2022) 400:1047–60. doi: 10.1016/S0140-6736(22)00879-0 36115372

[22] Gu Y You X Wang R. Workplace surface acting and employee insomnia: A moderated mediation model of psychological detachment and dispositional mindfulness. J Psychol. (2020) 154:367–85. doi: 10.1080/00223980.2020.1757595 32394806

[23] Sheng J Wang J. Correlation between sleep quality and anxiety and depression among clinical nurses in a tertiary care hospital. J Nurs. (2021) 22):16–8.

[24] Patel RM Bartholomew J. Impact of job resources and job demands on burnout among physical therapy providers. Int J Environ Res Public Health. (2021) 18:12521. doi: 10.3390/ijerph182312521 34886248 PMC8656566

[25] D'Ettorre G Pellicani V Caroli A Greco M. Shift work sleep disorder and job stress in shift nurses: implications for preventive interventions. Med Lav. (2020) 111:195–202. doi: 10.23749/mdl.v111i3.9197 32624561 PMC7809943

[26] American Psychology Association. The road to resilience: what is resilience [EB/OL] (2024). Available online at: http://www.Spa.Org/Helpcenter/Mad-Resilience.Aspx.

[27] Yu X Zhang J. Factor analysis and psychometric evaluation of the Connor-Davidson Resilience Scale (CD-RISC) with Chinese people. Soc Behav Personality: Int J. (2007) 35:19–30. doi: 10.2224/sbp.2007.35.1.19

[28] Li E. A study of the validity and reliability of the Insomnia Severity Index Scale. Guangzhou China: South China Medical University (2019). master's degree thesis (MSc).

[29] Wen Y Wu D Lv X Li H Liu X Yang Y. Evaluation of the reliability and validity of the Chinese condensed version of the Depression-Anxiety-Stress Scale. China Public Health. (2012) 11):1436–8.

[30] Erdoğan S Can AA Abiç A Yilmaz DV. Examination of individuals' depression, anxiety, and stress levels during the COVID-19 pandemic in Turkey. Arch Psychiatr Nurs. (2022) 41:96–102. doi: 10.1016/j.apnu.2022.07.021 36428081 PMC9307468

[31] Liu M Gu Y Jiang P Liu J Wu L Hu S. Current situation and influencing factors of negative emotions among obstetric nurses in Wuxi, China. Occup Health. (2021) 21):2933–7.

[32] Uccella S Mongelli F Majno-Hurst P Pavan LJ Uccella S Zoia C. Psychological impact of the very early beginning of the COVID-19 outbreak in healthcare workers: A bayesian study on the italian and swiss perspectives. Front Public Health. (2022) 10:768036. doi: 10.3389/fpubh.2022.768036 35400074 PMC8987285

[33] Khatony A Zakiei A Khazaie H Rezaei M Janatolmakan M. International nursing: A study of sleep quality among nurses and its correlation with cognitive factors. Nurs Adm Q. (2020) 44:E1–E10. doi: 10.1097/NAQ.0000000000000397 31789753

[34] Chung Y Kim H Koh DH Park JH Yoon S. Relationship between shift intensity and insomnia among hospital nurses in korea: A cross-sectional study. J Prev Med Public Health. (2021) 54:46–54. doi: 10.3961/jpmph.20.555 33618499 PMC7939760

[35] Bukowska-Damska A Reszka E Kaluzny P Wieczorek E Przybek M Zienolddiny S. Sleep quality and methylation status of core circadian rhythm genes among nurses and midwives. Chronobiol Int. (2017) 34:1211–23. doi: 10.1080/07420528.2017.1358176 29106308

[36] Meneo D Samea F Tahmasian M Baglioni C. The emotional component of insomnia disorder: A focus on emotion regulation and affect dynamics in relation to sleep quality and insomnia. J Sleep Res. (2023) 32:e13983. doi: 10.1111/jsr.13983 37394234

[37] Furihata R Saitoh K Suzuki M Jike M Kaneita Y Ohida T. A composite measure of sleep health is associated with symptoms of depression among Japanese female hospital nurses. Compr Psychiatry. (2020) 97:152151. doi: 10.1016/j.comppsych.2019.152151 31954287

[38] Vandekerckhove M Cluydts R. The emotional brain and sleep: an intimate relationship. Sleep Med Rev. (2010) 14:219–26. doi: 10.1016/j.smrv.2010.01.002 20363166

[39] Di Simone E Fabbian F Giannetta N Dionisi S Renzi E Cappadona R. Risk of medication errors and nurses' quality of sleep: a national cross-sectional web survey study. Eur Rev Med Pharmacol Sci. (2020) 24:7058–62. doi: 10.26355/eurrev_202006_21699 32633400

[40] Gupta A Love A Kilpatrick LA Labus JS Bhatt R Chang L. Morphological brain measures of cortico-limbic inhibition related to resilience. J Neurosci Res. (2017) 95:1760–75. doi: 10.1002/jnr.24007 PMC5512424 28029706

